# The Health Belief Model Modifying Factors Associated with Missed Clinic Appointments among Individuals with Sickle Cell Disease in the Jazan Province, Saudi Arabia

**DOI:** 10.3390/healthcare10122376

**Published:** 2022-11-26

**Authors:** Sami A. Alhazmi, Afnan Q. Maashi, Shahad K. Shabaan, Aisha A. Majrashi, Mawaeed A. Thakir, Safa M. Almetahr, Alanoud M. Qadri, Abdulaziz A. Hakami, Siddig I. Abdelwahab, Abdulaziz H. Alhazmi

**Affiliations:** 1 Faculty of Medicine, Jazan University, Jazan 45142, Saudi Arabia; 2 Medical Research Center, Jazan University, Jazan 45142, Saudi Arabia

**Keywords:** SCD, health belief model, clinic, complications, Jazan, Saudi Arabia

## Abstract

In treating chronic illnesses, such as sickle cell disease (SCD), outpatient care is essential; poor adherence in attending clinic appointments can lead to serious outcomes. SCD is highly prevalent in Saudi Arabia, and patients with SCD are advised to follow up with their treating physician in order to control this disease manifestation and to better forecast its complications. Studies evaluating missed appointments among patients with SCD are rare. Therefore, the current study aimed to use the health belief model’s modifying factors in order to evaluate the variables associated with poor adherence in attending appointments. A total of 381 participants with SCD from various regions in the Jazan Province, southwestern Saudi Arabia, were included. The survey instrument included socioeconomic determinants, factors associated with poor adherence in attending outpatient appointments, and solutions under the conceptual framework of the health belief model. A descriptive analysis was conducted and the factors that impacted adherence in attending the appointments were evaluated. In the current sample, respondents with SCD from 21 to 30 years represented 41%, which was followed by participants who were 11 to 20 years at 21.5%. In addition, about 60% of the participants were women. Further, approximately 62% of the patients admitted were missing one or more outpatient appointments in the previous year, which was significantly related to various factors, such as socioeconomic characteristics and patient residence. Forgetting the appointment was the main reason for skipping outpatient appointments for patients with SCD; as such, reminders appear to be a good solution for most participants. Our findings indicated that modifying components of the health belief model, including age, level of education, income, patients’ residence, and lacking cues to action (such as reminders) are important in explaining the reason for poor adherence in attending appointments. Thus, efforts are needed to address these factors and to ensure that SCD patients uphold their appointments. Future studies should examine the clinical, psychological, and epidemiological aspects that are linked with missed consultations.

## 1. Introduction

Sickle cell disease (SCD) is an inherited red blood cell disorder that is caused by the presence of a mutated form of hemoglobin, i.e., hemoglobin S (HbS). The prevalence of the birth of homozygous SCD is 112 per 100,000 live births worldwide. In addition, heterozygous SCD births are estimated at 4230 per 100,000 [1]. Sickle cell anemia is a common disease in Saudi Arabia. The Saudi Premarital Screening Program estimates the frequency of sickle cell anemia to be 5/1000 for the sickle cell trait and 0.38/1000 for the SCD itself. According to newborn screening, 2.6% of infants have the sickle cell illness and 21% possess the sickle cell trait [1]. Cerebrovascular accidents, acute chest syndrome, vaso-occlusive crises, and repeated infections are examples of significant medical complications that can occur as a result of SCD [2,3]. Therefore, in order to avoid complications and maintain a healthy and good quality of life, patients with SCD must obtain preventative care and adhere to follow-up appointments with their treating physicians [3]. Patients’ access to medical and pharmacological therapy relies on regular follow-ups with treating physicians, who can evaluate treatment benefits and side effects, as well as provide referrals for essential services [3].

Patients with SCD are advised to visit the clinic every six months or more if they experience frequent pain episodes and other symptoms [4]. In addition, immunization and other prophylaxis measures are provided as the standard of care during routine visits in order to avoid mentioned sequences of SCD [4,5,6]. Outpatient clinics reported a high rate of missed appointments that impaired proper medical management and wasted administrative and medical resources, which may be related to poor outcomes [7,8]. It could be the case that patients who do not adhere to scheduled appointments could represent one of the most costly problems in outpatient care in terms of economic and human resources [6].

The health belief model is one of the most well-established theoretical models that can be used to evaluate health promotion and disease prevention programs [9,10]. Therefore, this study aimed to assess the modifying factors of the health belief model associated with missed clinic appointments among individuals with SCD.

## 2. Materials and Methods

### 2.1. Study Area and Design

This study is a descriptive cross-sectional study conducted in the Jazan Province, located in the southwest corner of Saudi Arabia [11]. The province harbors almost two million inhabitants. Furthermore, the data were collected between May and July 2022.

### 2.2. Inclusion and Exclusion Criteria

We included patients with SCD of the male and female sex in the Jazan Province who had already been diagnosed with SCD and had been followed up with hematology clinics. We excluded individuals having other comorbidities, being followed outside of Jazan Province, and/or those who refused to participate.

### 2.3. Data Collection

**Figure 1:**
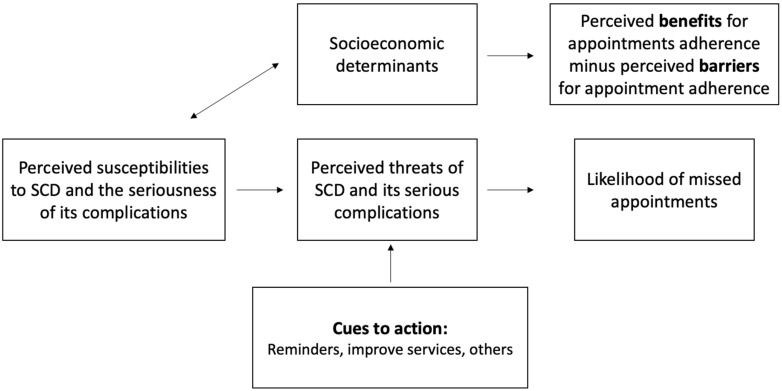
Health belief model for missed appointments.

Responses were gathered through a self-administered survey. The questions were constructed after a pretested questionnaire in accordance with our subjects and are intended to estimate modifying factors of the health belief model for patients with SCD in the Jazan area (Figure 1) [9,10].

### 2.4. Study Measures

The questionnaire includes questions that evaluate socio-demographics and included the modifying factors of the health belief model associated with missed clinic appointments among individuals with SCD. The questionnaire started with a question about whether the individual was already diagnosed with SCD. Then, those with positive answers would be directed to further questions about their age, sex, educational background, and job. Another part of the questionnaire was regarding whether the individual missed an outpatient appointment in the previous year. Then, those who answered yes were requested to answer questions about the reasons for the missed appointment, as well as solutions to avoid them.

### 2.5. Pilot Study

Before the distribution of the survey, a pilot sample (*n* = 20) was used to evaluate the clarity and wording of the questionnaire items, as well as to determine the internal consistency and external reliability of the survey using Cronbach’s alpha and test–retest analysis, which resulted in satisfactory scores (over 0.80). Data from this pilot sample were not included in the analysis.

### 2.6. Sampling Size and Statistical Analysis

In regard to the sample size, we assumed that there were about 4500 patients with SCD in the Jazan Province [1,11]. The sample size for this study was calculated using the Raosoft sample size calculator http://www.raosoft.com/samplesize.html (accessed on 1 May 2022) (Raosoft Inc., Seattle, WA, USA). Moreover, 351 patients with SCD were required in order to reach a 95% confidence interval and 5% margin of error. The completed questionnaires were edited for quality, consistency, and completeness before processing the results. The information was then coded. Then, data were subjected to a descriptive and univariate analysis using SPSS v23 (IBM Corp., Armonk, NY, USA), with the alpha criterion for the *p*-value set at 0.05.

### 2.7. Ethical Consideration

Ethical approval for conducting this study was obtained from the ethical approval committee at Jazan University (reference number: REC43/10/220; date: 9 May 2022). Written consent was obtained from all participants, or their guardians, before the study. The study was conducted following the ethical guidelines of the Helsinki Declaration and the local guidelines of the National Committee of Bioethics, SA. All collected data were kept confidential and used only for the purpose of this research. The questionnaires did not include the participants’ personal information or any other identification methods. Participants were given the right to continue or withdraw at any time from the study.

## 3. Results

We received 381 responses from patients with SCD, or from their tutors. The demographic data of the study, from Table 1, showed that 40% of the participants were between 21 and 30 years, followed by 11 to 20 years at 21%. Around 60% (226) of the total sample participants were female, while the remaining 40% were males. Regarding marital status, 258 respondents were single, 108 were married, and 15 were divorced or widowed. Concerning education level, most respondents were university students (38.6%). In regard to monthly income, 60% of the participants earned between 2 k and 10 k Saudi Riyals (SAR). The other demographic data are summarized in Table 1.

**Figure 2:**
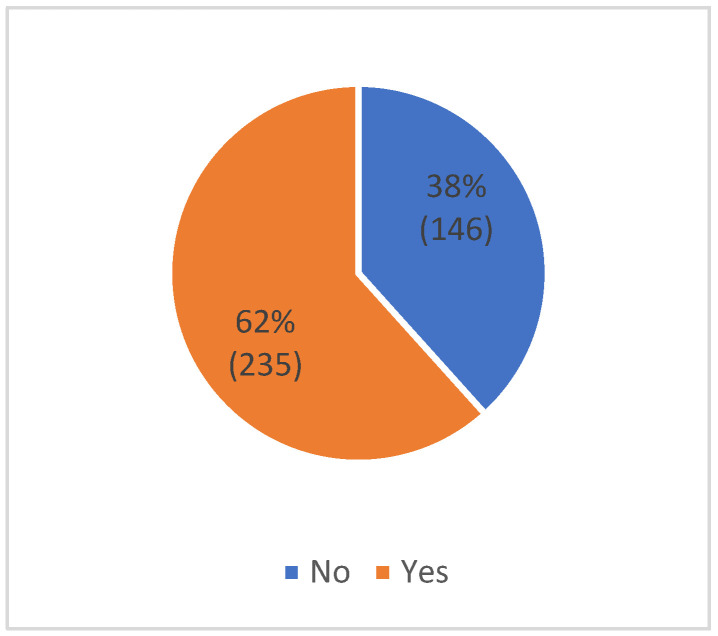
Participants who reported missing one or more appointments in the previous years.

**Figure 3:**
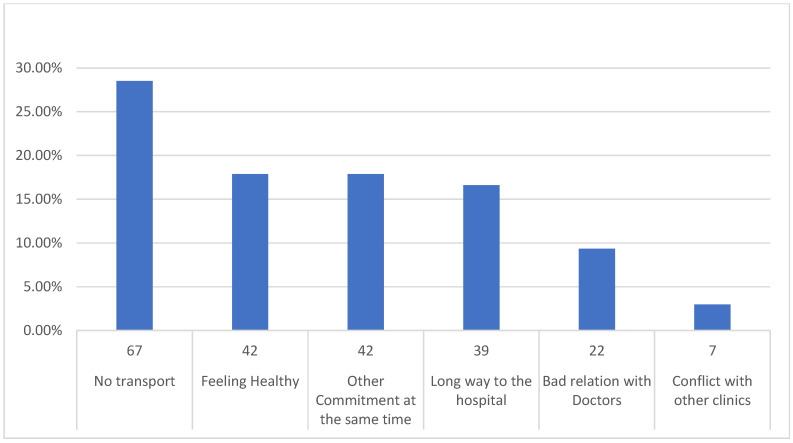
Reasons for missing clinical appointments among participants with SCD.

**Figure 4:**
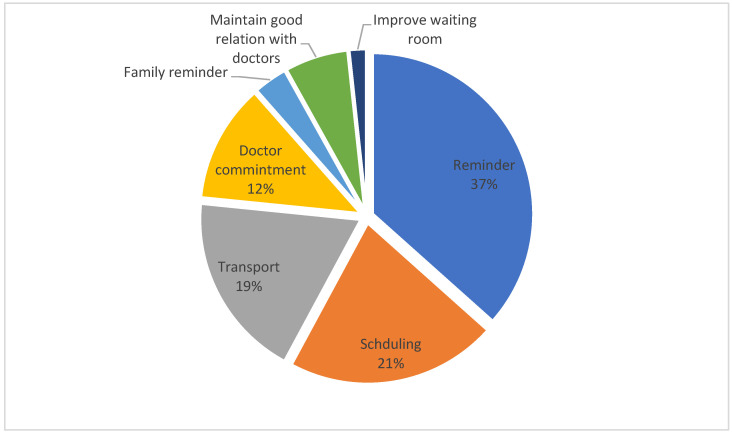
Factors that improve clinic attendance for participants with SCD.

According to the data presented in Figure 2, almost 62% (235) of patients diagnosed with SCD admitted that they had skipped one or more clinical visits. Figure 3 provides a summary of the many causes for this non-adherence and Figure 4 provides suggestions for how they may improve their level of adherence in attending the visits they have scheduled.

In Table 2, we compared factors associated with poor adherence in appointments against those who had better adherence in attending the appointment. We found a statistically significant relationship between missed clinic appointments and the respondents’ characteristics. Out of the total sample, 46% of respondents aged between 21 and 30 years reported more missed clinical appointments (*p*-value = 0.003). Then, higher education-level individuals reported missing clinical appointments (42% vs. 33%, *p*-value = 0.017). Regarding income, 33.2% of participants earning between 5 k and 10 k Saudi SAR missed more appointments than others (*p*-value = 0.022). Other factors are summarized in Table 2.

## 4. Discussion

SCD is a chronic disease that is highly prevalent in Saudi Arabia, including the Jazan province. We previously investigated how this disease could affect the affected individual’s quality of life and academic performance [1,12,13]. Adherence to appointments with the treating physician is crucial for the purposes of managing and anticipating complications that are commonly associated with this disease [3]. A significant correlation was previously observed between emergency department visits and missed outpatient appointments in a retrospective cohort study that was conducted and published in 2020, in Al Madinah Al Munawara, Saudi Arabia. The authors indicated that one missed appointment is associated with 1.92 times emergency department visits in a study that included 247 patients with SCD [14]. Further, several studies discussed the fact that patients with SCD may have socioeconomic disadvantages. In the context of Saudi Arabia, a previous study found that patients from families with a lower income are more susceptible to the complications associated with SCD, due to poor adherence in attending therapy or attending clinic visits [15,16,17]. Thus, various factors could affect the adherence in attendance of patients to clinic appointments and will, consequently, affect their quality of life. Therefore, in this project, we used modifying variables from the health belief model in order to evaluate factors associated with missed clinic appointments among individuals with SCD in the Jazan Province, as well as actions that could improve adherence in attending the scheduled appointments.

We found that 62% of the included subjects reported missing one or more clinic appointments per year with their treating physicians, which was significantly associated with socioeconomic factors, such as age, education, monthly income, and the residence of the patients (Figure 2, Table 2). An approximate rate of missing appointments among children with SCD was previously reported to be 65%, as well as 73% for both children and adults; these results were published in a study in 2018 by Cronin et al. in the United States, in which individuals from 18 to 25 years old tended to skip more appointments when compared to other age groups [9]. In the current study, we found that individuals aged 11 to 20 years and 21 to 30 acknowledged a higher rate of missing appointments than other groups (*p*-value = 0.003). This group experienced a transition from pediatric to adult clinics. This transition is usually associated with an unpreparedness for the different demands in the adult medical environment, such as booking appointments without the help of family or staff and the mismatch between pediatric and adult treatment [18]. Thus, we encourage treating physicians to be aware of this gap, as well as to consider that additional medical and psychological support for the affected individuals may result in better adherence. In addition, health officials should exploit the advances made in telemedicine in order to follow up with those who reside in remote locations, as we found participants’ residence to be a significant factor associated with adherence in attending outpatient appointments (Table 2) [19].

Forgetfulness was among the various reasons listed by the participants of the current study as a reason to skip a scheduled appointment (Figure 3). This finding is agreed by others [3,9,20,21]. Reminders were deemed helpful by 75% of participants in ensuring they attended appointments on time (Figure 4). They requested reminders most often through phone calls. However, text message reminders were favored for those aged 18 to 30 when compared to older persons (i.e., 63% for text and 57% for phone, respectively), showing that various age groups prefer different kinds of reminders. It was found in a systematic review and meta-analysis that when examining reminders for clinic visits, people who received alerts increased their adherence in attending clinic visits by 23%. In addition, multiple alerts were shown to be substantially more effective than single ones and audio notifications outperformed text messages [22]. Therefore, health officials may consider scheduled reminders with an option to reschedule the appointment, which may aid in improving adherence.

Financial instability appears to have been a significant factor in adults skipping appointments, individuals with SCD often missed appointments due to transportation challenges or because they could not afford transportation (Figure 3). Enhancing accessibility to appointments and funding-related fees may be critical in order to improve adherence in attending appointments. These results could be linked to the cognitive difficulties and susceptibility of individuals with SCD to psychological problems [23]. While depression has been linked to chronic, unexpected pain occurrences and psychosocial suffering due to SCD, it is acknowledged that cognitive impairment in people with SCD may increase the chance of missing appointments [24]. Thus, providers need to be aware that patients with SCD and cognitive impairment, as well as those with lower income and depressive symptoms, may need extra attention when it comes to enhancing outpatient clinic adherence in attendance, which will consequently reduce acute healthcare visits.

It is noteworthy that this study was conducted during the COVID-19 pandemic, a disease that directly impacts the clinical course of patients with SCD [25]. However, very few projects were undertaken to examine how this pandemic affected healthcare access for patients with SCD [26]. Moreover, it was shown that fewer patients sought medical care during the pandemic. However, prolonged hospitalization was noticed among individuals with SCD when diagnosed with COVID-19 [24,26]. These observations were explained by the tendency of the patients with SCD to avoid adhering to attending their routine follow-up in order to reduce the risk of SARS-CoV-2 transmission. When infected, the treating physicians tended to keep these patients longer as a precautionary measure in order to monitor the expected complications [26,27]. However, this study was conducted between May and July 2022, when all restrictive measures against COVID-19 were already alleviated in Saudi Arabia. The real impact of the COVID-19 pandemic cannot be neglected and, as a consequence, this should be considered when the findings of this study are interpreted.

There are few published studies that have discussed poor adherence in attending outpatient appointments among individuals with SCD at the national and international levels. Thus, this study would pave the way for further studies and actions, especially when it comes from a region with a high prevalence of SCD. Further, this study was based on a survey that was distributed to the patients, or their guardians, with SCD in hematology clinics and used an online platform in order to ensure access to more patients. As such, a non-response bias cannot be excluded due to the barrier of internet accessibility in some areas and the difficulties of using technologies among a certain demographic of the population. In addition, 38% of the participants were university students and this population may have other reasons for poor adherence in attending the appointments that were not included in the current methodology, as well as other psychological, social, and spiritual confounders that should also be considered. Thus, studies based on medical records with various demographic and clinical data could lead to finding other variables associated with poor adherence in attending appointments. In addition, although we succeeded in reaching the different regions in the Jazan province, variations in the number of responses could limit findings originating from this variable. However, we believe that we include real regional data of patients with SCD in the Jazan Province, as well as the patients’ viewpoints of their disease condition and how it could affect their adherence in medical care.

## 5. Conclusions

Our findings showed that various factors of the health belief model impacted the ability of individuals with SCD to adhere to attending clinic visits in the Jazan Province, Saudi Arabia. These factors were limited to socioeconomic determinants and patients’ residence. Forgetting appointments was the most common reason for the participants in this study to have poor adherence in attending outpatient appointments. In this regard, however, reminders seemed a good solution for most of them. The results of this study will serve as a foundation for further studies as they reflect the awareness and knowledge of patients with SCD and their families in Jazan Province, SA, regarding adherence in attending appointments. Further, future projects should develop ideas that exploit technologies and advancements in telemedicine in order to ensure better adherence in attendance and, therefore, a better quality of life for this vulnerable group.

## Figures and Tables

**Table 1:** Personal characteristics of the study participants (*n* = 381).

Variables		N	%
Age	Less than one year	4	1.0%
	1 to 10	62	16.3%
	11 to 20	82	21.5%
	21 to 30	155	40.7%
	31 to 40	55	14.4%
	More than 40	23	6.0%
Sex	Female	226	59.3%
	Male	155	40.7%
Marital Status	Single	258	67.7%
	Married	108	28.3%
	Divorce or Widow	15	3.9%
Education	None	28	7.3%
	Primary	66	17.3%
	Middle	40	10.5%
	High	95	24.9%
	University	147	38.6%
	Postgrad	5	1.3%
Employment status	Unemployed	291	76.4%
	Employed	84	22.0%
	Retired	6	1.6%
Monthly income in SAR	Less than 2 k	102	26.8%
	2 k to 5 k	111	29.1%
	5 k to 10 k	109	28.6%
	More than 10 k	59	15.5%
Living with	Parents	251	65.9%
	Relatives	27	7.1%
	Spouse	84	22.0%
	Alone	19	5.0%
Residence	Gizan	64	16.8%
	Sabya	44	11.5%
	Samtah	36	9.4%
	AbuArish	104	27.3%
	Damad	26	6.8%
	Alardah	23	6.0%
	AhadAlmasarah	22	5.8%
	Alhuerrath	8	2.1%
	Addarb	8	2.1%
	Alaidabi	7	1.8%
	Baish	17	4.5%
	Other	22	5.8%

SAR: Saudi Riyals.

**Table 2:** Comparison between individuals who reported missed clinic appointments and those with better adherence for attendance.

Missed Clinics		No (*n* = 146, 38%)		Yes (*n* = 235, 62%)		*p*-Value
Variables		N	%	*n*	%	
Age	Less than one year	3	2.1%	1	0.4%	0.003 *
	1 to 10	34	23.3%	28	11.9%	
	11 to 20	38	26.0%	44	18.7%	
	21 to 30	47	32.2%	108	46.0%	
	31 to 40	17	11.6%	38	16.2%	
	More than 40	7	4.8%	16	6.8%	
Sex	Female	81	55.5%	145	61.7%	0.240
	Male	65	44.5%	90	38.3%	
Marital Status	Single	98	67.1%	160	68.1%	0.676
	Married	40	27.4%	68	28.9%	
	Divorce or Widow	8	5.5%	7	3.0%	
Education	None	14	9.6%	14	6.0%	0.017 *
	Primary	36	24.7%	30	12.8%	
	Middle	17	11.6%	23	9.8%	
	High	30	20.5%	65	27.7%	
	University	48	32.9%	99	42.1%	
	Postgrad	1	0.7%	4	1.7%	
Employment status	Unemployed	117	80.1%	174	74.0%	0.285
	Employed	28	19.2%	56	23.8%	
	Retired	1	0.7%	5	2.1%	
Monthly income in SAR	Less than 2 k	46	31.5%	56	23.8%	0.022 *
	2 k to 5 k	40	27.4%	71	30.2%	
	5 k to 10 k	31	21.2%	78	33.2%	
	More than 10 k	29	19.9%	30	12.8%	
Living with	Parents	96	65.8%	155	66.0%	0.077
	Relatives	10	6.8%	5	2.1%	
	Spouse	30	20.5%	54	23.0%	
	Alone	10	6.8%	21	8.9%	
Residence	Gizan	38	26.0%	26	11.1%	0.006 *
	Sabya	17	11.6%	27	11.5%	
	Samtah	11	7.5%	25	10.6%	
	AbuArish	36	24.7%	68	28.9%	
	Damad	8	5.5%	18	7.7%	
	Alardah	5	3.4%	18	7.7%	
	AhadAlmasarah	6	4.1%	16	6.8%	
	Alhuerrath	0	0.0%	8	3.4%	
	Addarb	3	2.1%	5	2.1%	
	Alaidabi	3	2.1%	4	1.7%	
	Baish	10	6.8%	7	3.0%	
	Other	9	6.2%	13	5.5%	

SAR: Saudi Riyals. * The alpha criterion for the *p*-value was set to 0.05.

## Data Availability

Data is available upon request from the researchers.
